# Does mRNA structure contain genetic information for regulating co-translational protein folding?

**DOI:** 10.13918/j.issn.2095-8137.2017.004

**Published:** 2017-01-18

**Authors:** Jian-Rong Yang

**Affiliations:** Department of Biology, Zhongshan School of Medicine, Sun Yat-sen University, Guangzhou 510080, China

**Keywords:** Translational elongation rate, Protein folding, mRNA secondary structure, Codon usage bias

## Abstract

Currently many facets of genetic information are illdefined. In particular, how protein folding is genetically regulated has been a long-standing issue for genetics and protein biology. And a generic mechanistic model with supports of genomic data is still lacking. Recent technological advances have enabled much needed genome-wide experiments. While putting the effect of codon optimality on debate, these studies have supplied mounting evidence suggesting a role of mRNA structure in the regulation of protein folding by modulating translational elongation rate. In conjunctions with previous theories, this mechanistic model of protein folding guided by mRNA structure shall expand our understandings of genetic information and offer new insights into various biomedical puzzles.

## INTRODUCTION

With regards to the full contents of genetic information, the answer to this fundamental question in biology has been frequently updated as newly emerging techniques and growing data constantly challenge our existing understandings (Ramos & Laederach, 2014). On one hand, novel functions of non-coding genome are uncovered (ENCODE Project Consortium, 2012). On the other hand, our understandings of genetic information in coding regions have also extended beyond canonical schemes of how protein folding is regulated within cells.

Commonly known as "the second half of genetic code"(Kolata, 1986), a vast pool of information is required for ensuring correct folding of polypeptide into its native structure. However, little is known about how information stored in nucleotide sequence is transmitted from genome into polypeptide chain. According to the Central Dogma, messenger RNA is frequently targeted for searching regulatory signals for protein folding. Indeed, as evidenced by unequal usage of synonymous codons and its correlation with efficiency and/or accuracy of translational elongation (Gingold & Pilpel, 2011), mRNA molecule obviously contains more information than primary protein sequence. This logic and decades of genomic sequencing have elucidated the association between codon usage bias and protein structures (Spencer & Barral, 2012; Tsai et al., 2008).

Nevertheless, secondary structure of mRNA is often overlooked, probably due to a lack of scalable experiments for detecting RNA structure (Eddy, 2014) and the complexity of *in silico* prediction for the structure of ribosome-bound mRNA. Recently, this viewpoint has altered due to the latest technical innovations, especially ribosome profiling (Ingolia et al., 2009) and several high-throughput assays for mRNA secondary structure (Graveley, 2016). As a result, a novel regulatory role of mRNA structure on protein folding emerges.

In this review, current models of co-translational protein folding were reviewed for elucidating the generic molecular mechanism for its linkage to mRNA structure. Several preliminary studies that correlate computationally predicted mRNA structures with protein conformation shall be discussed. A major focus was placed upon regulatory signals in major mRNA coding sequences rather than a specific mRNA fragment such as translational ramp at 5' end (Tuller et al., 2010). Furthermore, biologically relevant interpretations of this regulation were offered. 

## GENETIC INFORMATION GUIDING PROTEIN FOLDING

The complexity of protein folding have been conventionally summarized as the Levinthal's paradox. It states that the number of possible conformations of a small protein (around 100 residues) was so large that it would require more time than the lifespan of universe (10^16^ seconds) to explore all possibilities and choose the native conformation (Zwanzig et al., 1992). How this astronomic eternity is reduced to biologically feasible range has been a long-standing puzzle of protein folding. As a theoretic milestone of protein folding, the Anfinsen's dogma, also known as thermodynamic hypothesis, suggests that protein conformation is solely determined by its amino acid sequence. In other words, assuming the validity of the Anfinsen's dogma, accurate protein folding requires no additional genetic input other than primary protein sequence. Nevertheless, multiple exceptions to the Anfinsen's dogma were detected later, including but not limited to prion (Fraser, 2014) and kinetically stable proteins (Xia et al., 2007). It has confirmed the existence of regulatory signals that guide protein folding, including *trans* factors such as chaperones and *cis* factors such as codon optimality (see below).

There are two major modes of protein folding. On the one hand, protein folding occurs after the entire coding sequence has been fully translated. Upon dissociation of mRNA with synthesized polypeptide, a generic molecular mechanism of conveying regulatory signals in mRNA into a remote polypeptide chain is unlikely, if not impossible. On the other hand, nascent polypeptide folds co-translationally while it is being synthesized. Many reports suggested that folding of many proteins was at least partially co-translational (Hardesty et al., 1999; Pechmann et al., 2013; Rodnina & Wintermeyer, 2016). More importantly, mRNA, ribosome and nascent polypeptide form a complex, allowing the transmission and/or realization of regulatory signals in mRNA for protein folding (Pechmann et al., 2013). In fact, current models linking mRNA structure to protein folding are all based upon co-translational folding pathway. Here the discussion of post-translational folding is skipped and only co-translational folding highlighted.

## CONCEPTS OF CO-TRANSLATIONAL PROTEIN FOLDING PROCESS AND ITS REGULATION

Preliminary evidence of co-translational folding for at least some proteins appeared around the same time as Anfinsen performed his seminal experiments on ribonuclease (Cowie et al., 1961; Kiho & Rich, 1964). In theory, full-length unfolded polypeptide was energetically unfavorable (Fedorov & Baldwin, 1997) so that the folding process of most proteins should be more or less co-translational. How co-translational folding proceeds is another complicated point. As for the site of co-translational folding, there are two major steps of co-translational folding. Firstly, newly synthesized polypeptide had to travel through a ribosomal exit tunnel of approximately 80 angstrom in length (Fedyukina & Cavagnero, 2011), which is wide enough to accommodate α-helix formation. Indeed, α-helix within exit tunnel has been directly confirmed by FRET (fluorescence resonance energy transfer) (Woolhead et al., 2004). Additional evidence suggested that exit tunnel could entropically stabilize both α-helix (Ziv et al., 2005) and distinct conformations of nascent polypeptide via extensive contacts with ribosomal components (Bhushan et al., 2010). Secondly, other steps of co-translational folding, especially the higher order ones impossible within confined space of exit tunnel, occurred after a partial exit of polypeptide from ribosome. For example, co-translational folding of cystic fibrosis transmembrane conductance regulator was dissected experimentally (Kim et al., 2015). And its α-subdomain compaction was delayed until all related polypeptides migrated into cytosol (Kim et al., 2015).

Consistent with our understandings of co-translation protein folding, many *cis* and *trans* regulators have been implicated. Some discovered *trans* regulators include ribosome-bound chaperones capable of operationally extending exit tunnel and providing additional space for protein folding (Kramer et al., 2009). Also co-translational recognition by signal recognition particle (SRP) induced a translocation of nascent peptide into endoplasmic reticulum with a distinct folding environment (Pechmann et al., 2014). As for *cis* regulators, two distinct and yet probably synergistic mechanisms affect co-translational folding (Pechmann et al., 2014). One mechanism operates by recruiting certain *trans* regulators through specific motifs such as SRP-binding elements (Pechmann et al., 2014) while another by modulating elongation speed (O'Brien et al., 2014, 2012). Two mRNA features have been implicated in modulating elongation speed and thus regulating co-translational protein folding, without altering peptide sequences, i.e. codon optimality (Pechmann et al., 2014; Zhang et al., 2009) and mRNA secondary structures (Faure et al., 2016; Jia et al., 2004; Liu & Liu, 1999; Zhang et al., 1998).

## IMPACT OF CODON OPTIMALITY ON PROTEIN FOLDING

It was known that 18/20 amino acids are encoded by two or more synonymous codons. Among them, some are called "optimal" because of either their higher thermodynamic stability after pairing with anticodon or a higher abundance of their cognate tRNAs. The current mechanistic model of translational elongation dictates that codon optimality influences translational efficiency and/or accuracy (Gingold & Pilpel, 2011), i.e., optimal codons are translated faster (see below) and/or with higher fidelity. Unlike the sparse data for mRNA structure, accumulation of sequenced ORFs facilitated codon optimality profiling in a wide array of genes and species since 1970s, resulting in a large body of work investigating codon optimality-dependent modulation of translational elongation rate and its effect on co-translational protein folding. For example, Escherichia coli multidomain protein SufI was examined. Severe perturbation was reported for SufI folding efficiency by excessive tRNA *in vitro* or synonymous substitution into some clusters of non-optimal codons. It was assumed that the clusters of non-optimal codons transiently attenuated translational elongation, temporally separated the translation of segments of peptide chain and actively coordinated co-translational folding. Considering tRNA supply and demand, Pechmann and colleagues (Pechmann & Frydman, 2013) modeled efficiency of translational elongation in 10 closely related yeast species, and found evolutionarily conserved distribution of codon optimality that is associated with secondary structure of translated polypeptides. The authors suggested that mRNA sequences, and in particular synonymous codon choices, are generally under selection to optimize the co-translational folding of corresponding polypeptides. Altogether, these and other reports (Komar, 2009) have hinted at an evolutionarily conserved link between clusters of non-optimal codons and pauses of translational elongation that facilitates co-translational protein folding, and more importantly, the existence of additional genetic information in an ORF beyond primary protein sequence. Nevertheless, the exact molecular mechanism for such regulatory effect remains elusive, as cluster of non-optimal codons could have been evolved due to selection for sequence features other than the non-optimality of codons.

## MESSENGER RNA STRUCTURE AFFECTS TRANLATIONAL ELONGATION

As a critical component of genetic information flow for a certain protein coding gene, messenger RNA extracts coding sequences from genome and applies it as a template for protein synthesis. Nevertheless, not merely a sequence of codons, mRNA has its own complex structures. In particular, its secondary structure of Watson-Crick pairing between nucleotides could regulate translational processes at multiple levels. For example, stable secondary structure at the 5' end of mRNA might suppress translational initiation and thus enhance overall translation efficiency in* E. coli *(Kudla et al., 2009). Excessive stable stem regions at the 3' UTR decreased the accessibility of miRNA response elements and interfered with miRNA-mediated translational repression (Kertesz et al., 2007). While the above examples were limited to either end of mRNA, other functional roles of mRNA secondary structure have been discovered for major coding sequences, such as regulating RNA editing (Nishikura, 2006) and splicing (Shepard & Hertel, 2008). Furthermore, secondary structure of nascent mRNA might lower local mutation rate (Chen et al., 2016). More importantly, specific mRNA structure could interfere with the movement of translating ribosome (Brierley et al., 1991; Chen et al., 2013; Qu et al., 2011; Wen et al., 2008). Given numerous reports (Ciryam et al., 2013; O'Brien et al., 2014, 2012; Wang et al., 2015) connecting translational elongation rate to co-translational folding, it is thus not surprising that theoretical studies have already linked mRNA secondary structure to protein folding.

## EARLY EVIDENCE FOR THE REGULATORY ROLE OF mRNA STUCTURE ON PROTEIN FOLDING

The earliest report correlating mRNA structure with the regulation of protein folding appeared in 1993 (Guisez et al., 1993). In the study of Guisez et al., several nascent polypeptide intermediates of coat protein of RNA bacteriophage MS2 were analyzed. And the sizes of nascent polypeptide intermediates were found corresponded to either the positions of rare codons or RNA regions with double-stranded secondary structures, both presumably decrease the velocity of translating ribosomes. It was thus hypothesized that discontinuous translational elongation rate generally facilitates optimal folding of polypeptides. The hypothesized regulated protein folding by mRNA structure was later supported by two additional empirical analyses. On the one hand, the codons of hydrophobic and hydrophilic amino acids tend to respectively located in stem and loop regions of mRNA (Zhang et al., 1998). Given the crucial role of hydrophobic effect on stabilizing protein structure, such observation is suggestive for the information transfer between mRNA and protein structure (Zhang et al., 1998). On the other hand, experimentally determined protein secondary structures were directly compared with computationally predicted mRNA secondary structures (Jia et al., 2004). And α-helices and β-strands within a folded protein tend to be encoded by double-stranded mRNA regions whereas random coils within polypeptide were more likely to be encoded by single-stranded mRNA regions. Although these studies offered preliminary evidence for an intriguing link between mRNA and protein structure, their limitations were also obvious. On the one hand, due to the scarcity of experimentally determined mRNA structure, these studies resorted to computationally predicted mRNA secondary structure with a modest accuracy at best (Lange et al., 2012), let alone higher level structures. On the other hand, the proposed link between mRNA and protein structure was mediated by the capability of mRNA structure in modulating translational elongation speed, whose exact nature was mostly unknown by then. Recent genomic advances have enabled assessments of the above link.

## NOVEL GENOMIC DATA QUESTION THE REGULATION OF PROTEIN FOLDING BY CODON OPTIMALITY

The advances of high throughput sequencing techniques have allowed experimental explorations for both translational elongation speed and mRNA structure at the genomic levels (Graveley, 2016; Ingolia et al., 2012). And the resulting datasets have triggered empirical tests of two major hypotheses for *cis*-regulatory signal in mRNA for co-translational folding, i.e. codon optimality and mRNA structure (Qian et al., 2012; Tuller et al., 2011; Yang et al., 2014). Overall, the effect of codon optimality failed to receive consistent supports. The rationales for the regulatory role of mRNA structure in protein co-translational folding and its relationship with codon optimality shall be summarized in the next section.

Essentially as a snapshot for the distribution of ribosomes within transcriptome, ribosome profiling (Ingolia et al., 2009) utilized high-throughput sequencing of segmental mRNA shielded by translating ribosomes from endonuclease digestion, Since translating ribosomes spend more time on stretches of nucleotides with higher coverage in ribosome profiling than other nucleotides in the same gene, such detail ribosomal kinetics allowed revelation/confirmation of several critical features of translational elongation. Firstly, translational elongation rate was not uniform among different mRNAs or along a single mRNA molecule (Ingolia et al., 2009, 2011). Secondly, strong ribosomal pauses lasting over a couple of seconds, >10 times slower than average elongation speed, were widely distributed (Ingolia et al., 2011). Thirdly, at least some variations of elongation speed within gene was obviously non-neutral and had evolved under natural selections (Tuller et al., 2010). All the above findings were consistent with the model of co-translational protein folding as regulated by translational elongation rate, necessitating the validation of codon optimality and/or mRNA structure as a regulator of elongation rate. 

Indeed existing data of ribosome profiling have enabled independent assessments of the role of codon optimality in the control of translational elongation speed. Unexpectedly, several attempts of confirming the slow translational speed of individual non-optimal codons failed to reveal any signal in genomic ribosome profiling data of several species, including yeast (Charneski & Hurst, 2013; Qian et al., 2012), worms (Stadler & Fire, 2011), rodents (Ingolia et al., 2011) and bacteria (Li et al., 2012). These studies explicitly tested the correlation between codon optimality and elongation speed, and found negative results so that other determinants of elongation speed were examined, such as positively-charged amino acids (Charneski & Hurst, 2013), wobble base-pairing (Stadler & Fire, 2011) and anti-Shine-Dalgarno sequence (Li et al., 2012). In one of these studies, observations were explained by balanced synonymous codon usage of transcriptome relative to the abundance of tRNA (Qian et al., 2012). When there was an overall shortage of translation-ready tRNAs, balanced codon usage makes tRNA shortage for all codons similar. It avoided long ribosomal pauses caused by extreme tRNA shortage for a few codons and thus minimizing transcriptome-wide total duration of ribosomal pauses. Further analyses confirmed such a balanced codon usage for multiple eukaryotic transcriptomes. It hinted at adaptive evolution towards balanced codon usage, which presumably provides optimal allocation of translational resources and alleviated ribosomal sequestering due to translational pauses. As experimentally validated, global translational efficiency increased after a heterologous gene with balanced codon usage was transfected into yeast cells, compared to a gene using only optimal or non-optimal codons. The experimental observations were consistent with codon harmonization (Angov et al., 2008), a strategy commonly employed to enhance heterologous protein expression in synthetic biology. More importantly, the model of balanced codon usage indicates that previous experimental results correlating non-optimal codons with halt of translational elongation could be artefactual since most of them involved transfecting a highly expressed heterologous gene into a host cell. A high expression of heterologous gene perturbed the balance between codon usage and tRNA supply. Since the absolute number of cognate tRNA for optimal codon was higher, tRNA shortage was thus proportionally less serious for optimal than non-optimal codons so that there was faster translational elongation for optimal codons in heterologous system. Collectively, the above results cast doubts over the conventional wisdom of faster translation of optimal codons (Charneski & Hurst, 2013; Ingolia et al., 2011; Qian et al., 2012; Yang et al., 2014).

Nevertheless, the results suggesting no correlation between codon optimality and ribosomal velocity are not without their own problems. Most notebly, when cycloheximide was used for stabilizing ribosomes prior to position measurements, elongation re-occurred in the presence of cycloheximide but with dramatically altered codon-specific elongation rates. And the measured positions of ribosomes failed to reflect the temporal durations of ribosomal pausing at each position *in vivo* (Hussmann et al., 2015). Meanwhile, other studies have independently examined the correlation between codon optimality and translational elongation rate, but inconsistent results were obtained (Gardin et al., 2014; Li et al., 2012; Stadler & Fire, 2011). After analyzing multiple datasets of ribosome profiling, it was found that, regardless of using cycloheximide or not prior to cell lysis, the reproducibility of ribosome profiling was poor at codon resolution since signals at this level were not well-reproduced in experimental replicates (Diament & Tuller, 2016). Previous theoretical and experimental results have confirmed the regulation of co-translational protein folding by clusters of non-optimal codons through modulation of elongation speed. However, the exact molecular mechanism for non-optimal codon cluster stalling ribosomal movement has remained elusive. It left the possibilities of alternative or synergistic regulations other than codon optimality, such that the clusters of non-optimal codons are probably byproducts of other sequence features.

## NOVEL GENOMIC DATA SUPPORTING THE REGULATION OF PROTEIN FOLDING BY mRNA STRUCTURE

The development of high-throughput sequencing has enabled multiple methods for examining RNA secondary structure at a genomic level. Early attempts of FragSeq (Underwood et al., 2010), PARS (Kertesz et al., 2010) and SHAPE-seq (Lucks et al., 2011) utilized P1 nuclease, RNase V1 & S1 nuclease and 1-methyl-7-nitroisatoic anhydride respectively, to probe the structures of a large pool of synthetic RNAs or total RNA after extraction from cells, which revealed *in vitro* pairing status of individual nucleotides on RNA molecules. These approaches were followed by the development of mod-seq (Talkish et al., 2014), DMS-seq (Rouskin et al., 2014), Structure-seq (Ding et al., 2014), icSHAPE (Spitale et al., 2015) and SHAPE-Map (Smola et al., 2015), which were capable of detecting* in vivo* RNA secondary structure. More recently, techniques have been developed for detecting pairing partners, including RPL (Ramani et al., 2015), PARIS (Lu et al., 2016), SPLASH (Aw et al., 2016) and LIGR-seq (Sharma et al., 2016). These advanced techniques have cleared the obstacles of genomic RNA structure investigation and enhanced the profiling accuracy of RNA secondary structure (Lange et al., 2012).

Combined with ribosomal profiling data, genomic profiles of RNA secondary structure supported the role of mRNA structure in modulating elongation speed (Tuller et al., 2010, 2011; Yang et al., 2014) (but see Charneski & Hurst, 2013). In particular, comparisons among genes revealed stronger mRNA secondary structure (Zur & Tuller, 2012) and slower translational elongation (Yang et al., 2014) for highly expressed genes, which is more sensitive to protein misfolding (Yang et al., 2010; Zhang & Yang, 2015). Additional comparisons within gene revealed that mRNA pairing status at the entrance of ribosome had the strongest impact upon elongation rate (Yang et al., 2014). This result was consistent with single molecule level study of individual translating ribosome using optical tweezers, who found that pausing duration of ribosomal translocation was significantly dependent on mRNA secondary structure (Wen et al., 2008). More importantly, the regulatory effect of mRNA secondary structure on ribosome velocity seemed independent of codon optimality (Yang et al., 2014). Thus mRNA secondary structure might serve as a regulator of protein co-translational folding via modulating elongation speed. During translation, mRNA secondary structures were actively unfolded by ribosomes (Rouskin et al., 2014). However, the distance between adjacent ribosomes was approximately 20-35 nm in eukaryotes. And it was translated into 50-90 nt or 17-30 codons, allowing enough time for intervening mRNA to refold given the thousand fold difference between timescale of ribosomal elongation (~0.1s per codon (Ingolia et al., 2009, 2011) and RNA folding kinetics (10^-5^ s for folding a simple hairpin (de Smit & van Duin, 2003)). 

More recently, new genomic data were used for directly testing the connection between mRNA structure and protein folding by comparison among genes (Faure et al., 2016). Using protein structures from 2 eukaryotes and 3 prokaryotes, protein compactness was positively correlated with the stability of mRNA structure. Such correlations are more pronounced in ordered parts than disordered parts of protein. Thus it suggested an important role of mRNA secondary structure in modulating protein folding. More importantly, comparison with translational efficiency inferred from ribosome profiling data supported that stable mRNAs were translated slowly to allow more time for compact proteins to fold co-translationally (Faure et al., 2016).

**Figure 1:**
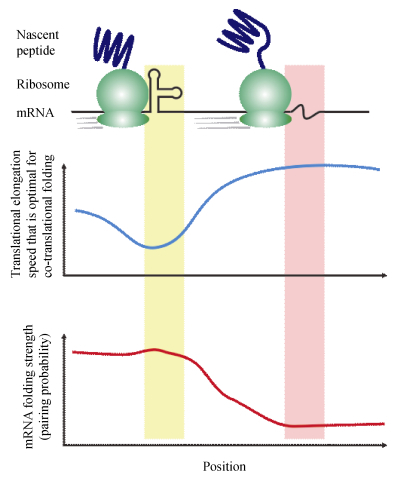
A mechanistic model of co-translational protein folding under the regulation of mRNA secondary structure

Collectively, new genomic data of ribosome profiling and mRNA secondary structure suggested a mechanistic model (Fig. 1), where the co-translational protein folding is regulated by mRNA secondary structure through its modulation of translational elongation rate. Such a regulation was independent of elongation slowdown due to nonoptimality of synonymous codon usage, whose capacity of regulating co-translational protein folding has remained debated. 

## BIOMEDICAL IMPLICATIONS

In conclusion, elucidating the regulatory role of mRNA secondary structure for protein folding shall expand our understandings of the full contents of genetic information and the molecular mechanisms of its phenotypic expression. Gaining such insights offers broad implications for biological researches. For example, combined with proper bioinformatic algorithm for designing mRNA structure, it is bound to enhance our capability of expressing functionally heterologous proteins in cells. The generality of this regulatory role has raised questions on the neutrality of synonymous variations in coding sequences for molecular evolutionary analyses. The regulatory role of mRNA structure in protein folding has become the only model capable of explaining the stronger mRNA secondary structures in highly expressed genes (Yang et al., 2014; Yang & Zhang, 2015). It offers a fundamental tool of understanding how natural selection concerts the optimality of synonymous codon usage and mRNA secondary structure and subsequently affects the evolution of coding sequences. Co-translational misfolding is a form of phenotypic mutation. The regulatory role of mRNA structure in both protein folding and mutation rate may lead to a quantitative coupling between genotypic and phenotypic mutation rates (Chen et al., 2016). Full biological ramifications of such intriguing coupling between processing and transmission fidelity of genetic information await further explorations.

Detailed modeling of co-translational folding modulated by mRNA structure can help us predict or interpret the phenotypic effects and elucidate the underlying mechanisms of synonymous variations ubiquitous in human genome (The 1000 Genomes Project Consortium, 2010). Implicated in human diseases (Kimchi-Sarfaty et al., 2007), it is considered as frequent driver mutations in human cancer (Supek et al., 2014). Altered mRNA structure might result in a dysregulation of co-translational protein folding, leading to protein misfolding and aggregation that is disproportionately involved in neurodegenerative diseases (Soto, 2003). Understanding the subtle roles of mRNA structure in protein misfolding and aggregation shall reveal new therapeutic targets for neurodegenerative diseases.

## ACKNOWLEDGEMENTS

Great appreciations were extended to Yong-Gang Yao, Gong-Wang Yu, Heng Zhang and two anonymous reviewers for valuable comments. 
